# Supplementary figures and images for: Xylella fastidiosa subsp. pauca and olive produced lipids moderate the switch adhesive versus non-adhesive state and viceversa
Source: PLoS One. 2020 May 15;15(5):e0233013. doi: 10.1371/journal.pone.0233013 (PMC7228078; doi:10.1371/journal.pone.0233013)

**S1 Fig.**


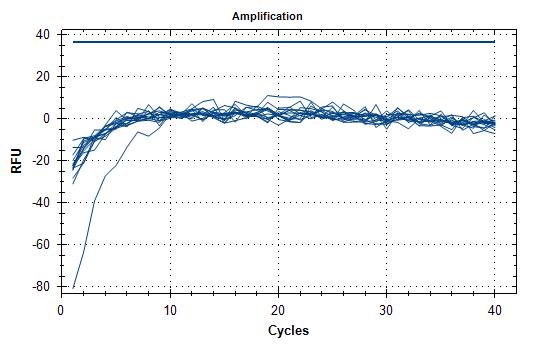

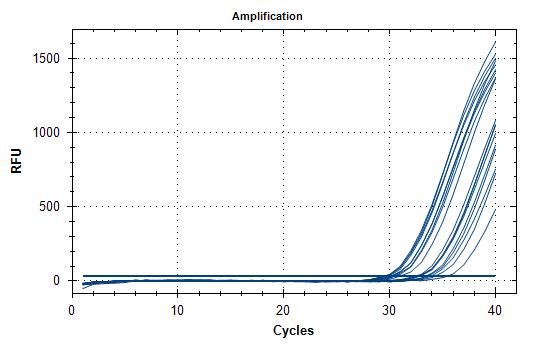


**A) B)**

Supplement: S1 Fig — A) Amplification curves of real-time PCR of OQDS- samples and B) of OQDS+ samples following the protocol published by Harper and colleague [29]. (DOCX) [file pone.0233013.s001.docx]

**S2 Fig**

**
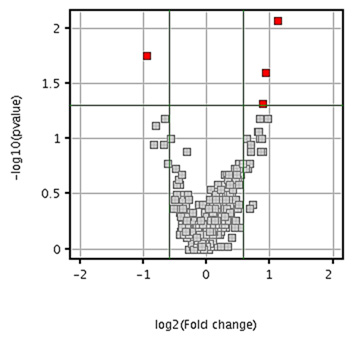
**

Supplement: S2 Fig — The x-axis shows the fold change to indicate the variation in abundance of compounds present in Xf+ compared to Xf-. The compounds on the right side were more abundant, whereas those on the left side were less abundant in Xf+ condition. Entities that satisfied the fold change and the P-value cut-off of 1.5 and 0.05, respectively, are marked in red. (DOCX) [file pone.0233013.s002.docx]

**S3 Fig**

**
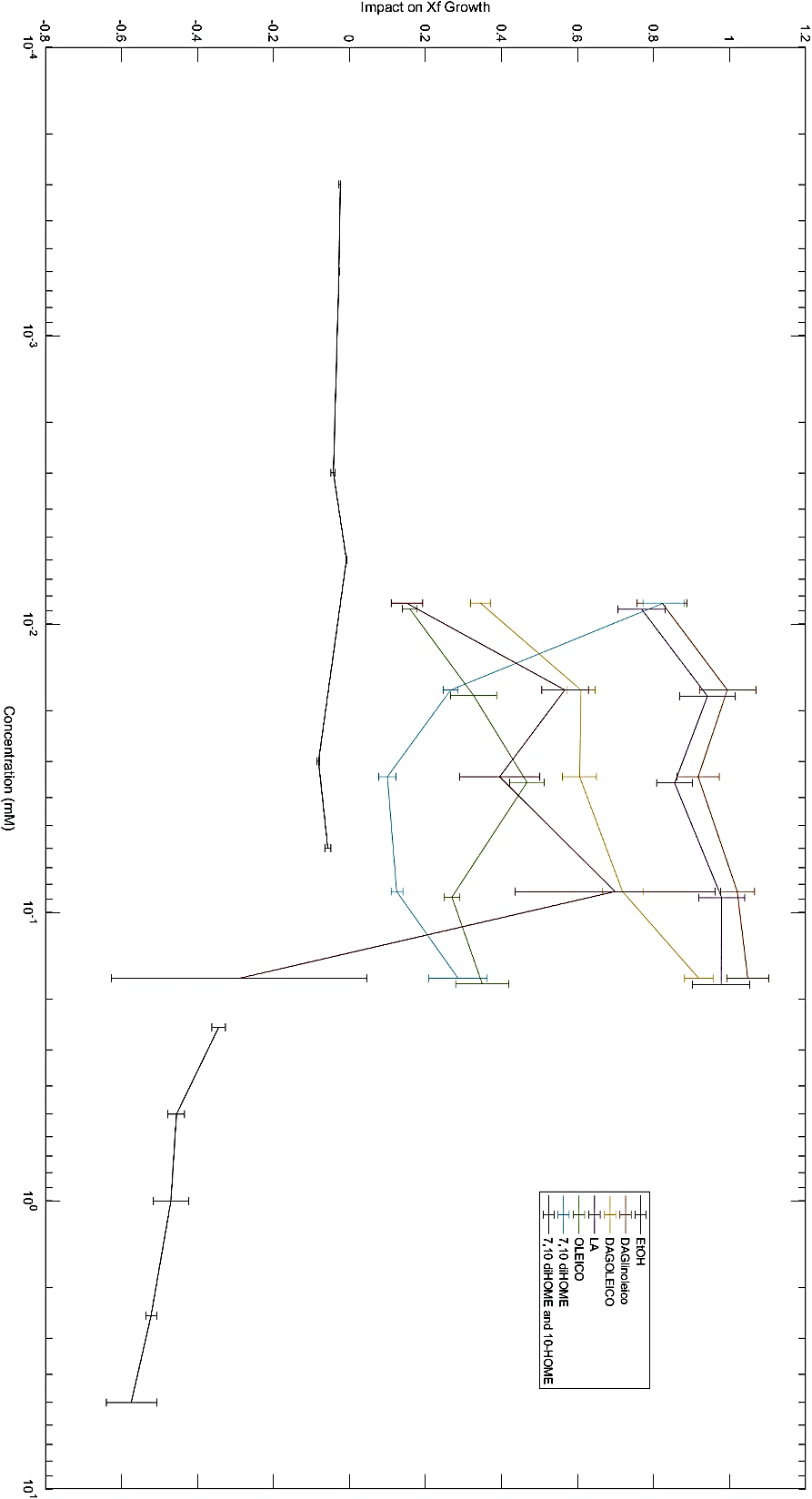
**

Supplement: S3 Fig — Impact is expressed as OD600 fold-change compared to the control, the effect of the corresponding amounts of ethanol employed to dissolve the compounds has been subtracted. Concentrations are expressed in molarity. The effect of ethanol at equivalent and higher molarities is plotted as a reference. Tested compounds were evaluated through Kruskal Wallis test and classified by Fisher LSD post-hoc test with Bonferroni correction (p-value<0,05). (DOCX) [file pone.0233013.s003.docx]

**S4 Fig**

**
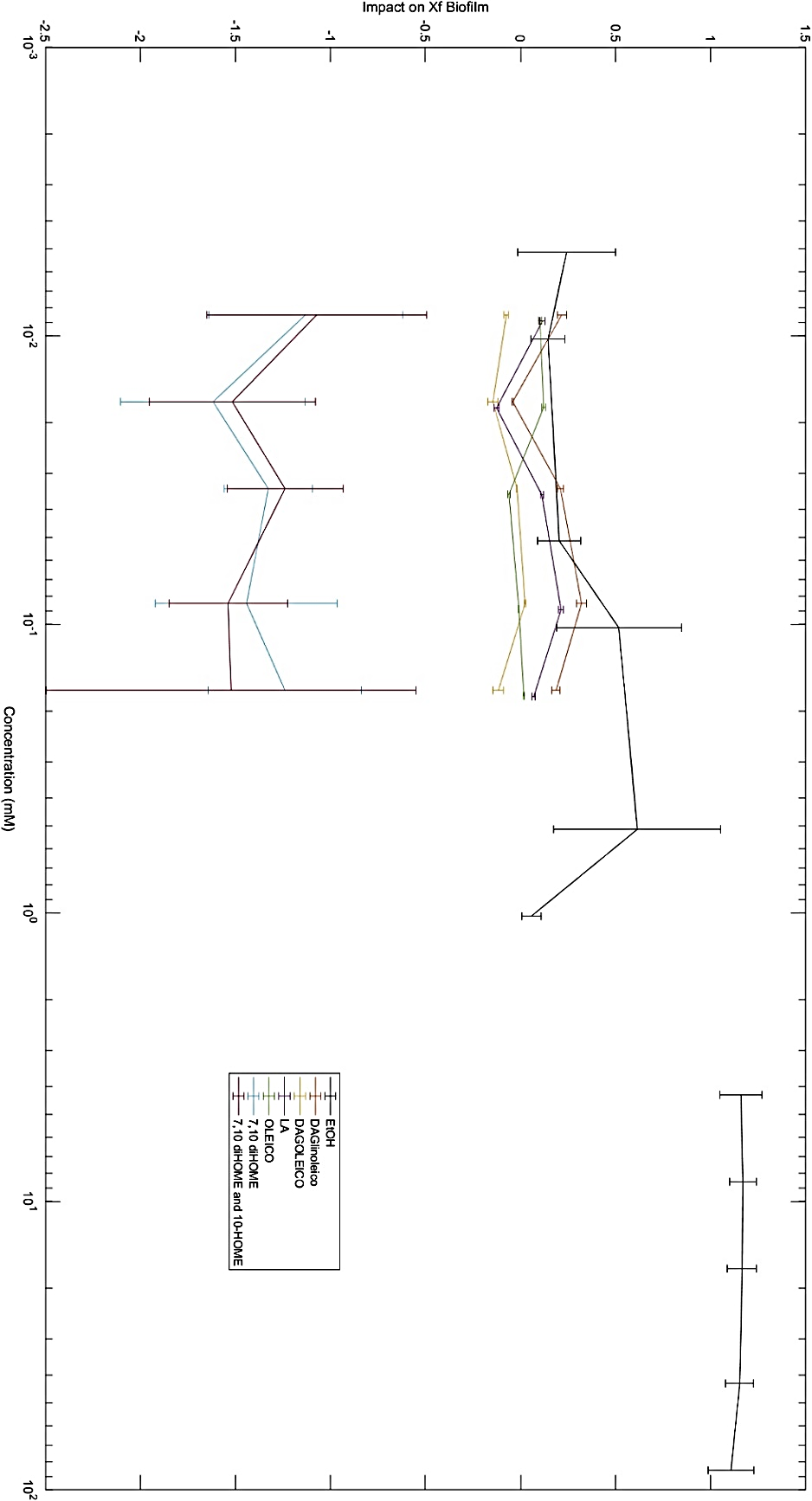
**

Supplement: S4 Fig — Impact is expressed as OD595 fold-change compared to the control. Concentrations are expressed in molarity. The effect of ethanol at equivalent and higher molarities is plotted as a reference. Tested compounds were evaluated through Kruskal Wallis test and classified by Fisher LSD post-hoc test with Bonferroni correction (p-value<0,05). (DOCX) [file pone.0233013.s004.docx]

**S5 Fig**

**
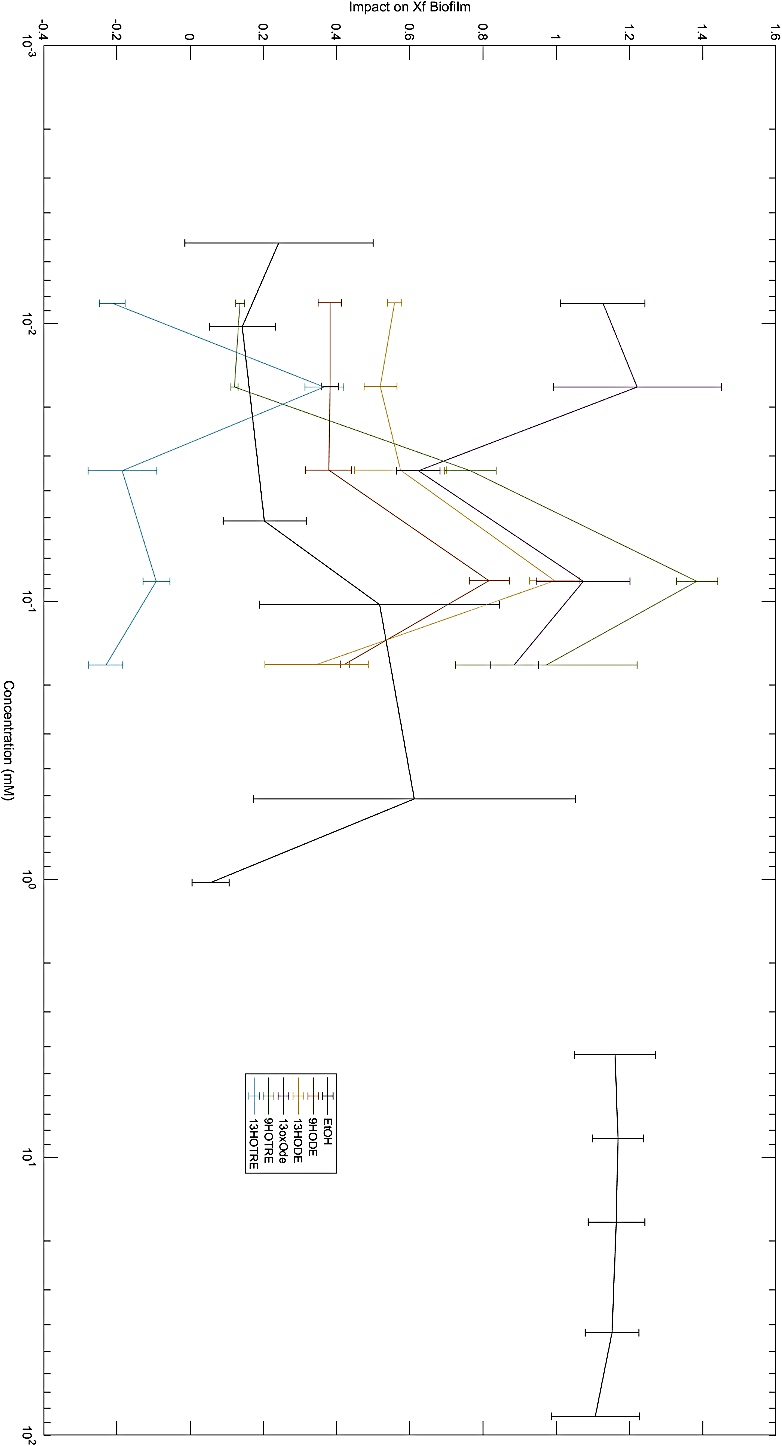
**

Supplement: S5 Fig — Impact is expressed as OD595 fold-change compared to the control, the effect of the corresponding amounts of ethanol employed to dissolve the compounds has been subtracted. Concentrations are expressed in molarity. The effect of ethanol at equivalent and higher molarities is plotted as a reference. Tested compounds were evaluated through Kruskal Wallis test and classified by Fisher LSD post-hoc test with Bonferroni correction (p-value<0,05). (DOCX) [file pone.0233013.s005.docx]

**S6 Fig**

**
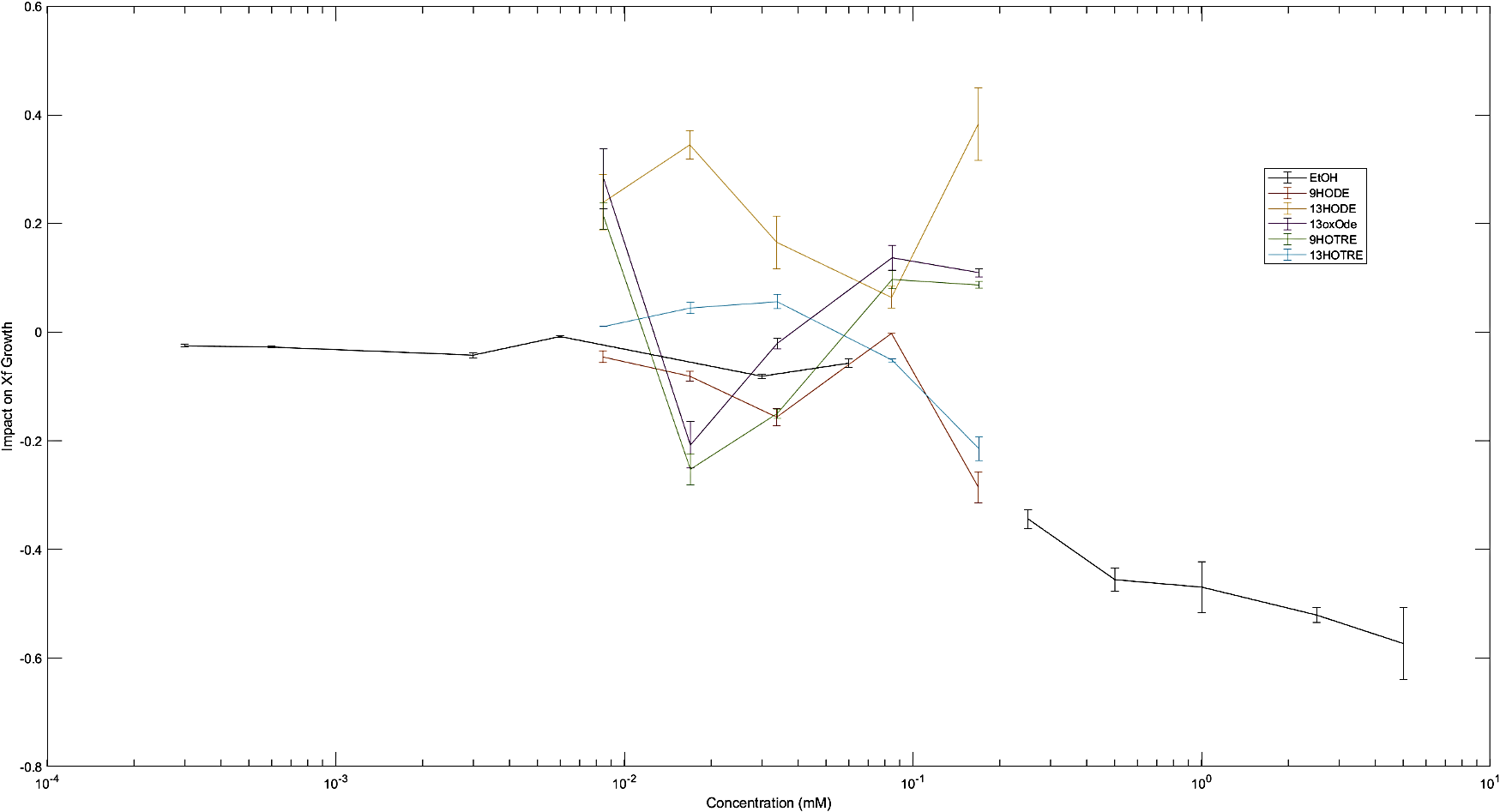
**

Supplement: S6 Fig — Impact is expressed as OD600 fold-change compared to the control, the effect of the corresponding amounts of ethanol employed to dissolve the compounds has been subtracted. Concentrations are expressed in molarity. The effect of ethanol at equivalent and higher molarities is plotted as a reference. Tested compounds were evaluated through Kruskal Wallis test and classified by Fisher LSD post-hoc test with Bonferroni correction (p-value<0,05). (DOCX) [file pone.0233013.s006.docx]
