# Supplementary material for: Xylella fastidiosa subsp. pauca and olive produced lipids moderate the switch adhesive versus non-adhesive state and viceversa
Source: PLoS One. 2020 May 15;15(5):e0233013. doi: 10.1371/journal.pone.0233013 (PMC7228078; doi:10.1371/journal.pone.0233013)
Supplement: S1 Table — A) MRM analysis method for oxylipins; B) MRM method for phospholipids, glycerolipids, ornitholipids, bactophenols; C) SIM method for free fatty acids. (DOCX) [file pone.0233013.s007.docx]

**S1 Table.**

**A**

| Analyte | Transition | Fragmentor (V) | Collision energy (eV) |
| --- | --- | --- | --- |
| jasmonate | 209,2→59,1 | 135 | 28 |
| methyl-jasmonate | 223,1→75,2 | 135 | 25 |
| 9 - HODEd4 (ISTD) | 299,2→172,2 | 140 | 20 |
| 12,13 - diHOME | 313,3→183,1 | 140 | 18 |
| 9,10 - diHOME | 313,3→201,1 | 140 | 14 |
| 8,13 - diHODE | 311,3→171,2 | 140 | 14 |
| 9 – HOTrE | 293,2→171,2 | 140 | 18 |
| 13 - HOTrE | 293,2→195,2 | 140 | 18 |
| 10-HOME | 297,2→155,2 | 135 | 36 |
| 9-oxoOTrE | 291,4→185,2 | 140 | 18 |
| 8 – HODE | 295,2→157,2 | 140 | 20 |
| 13 – HODE | 295,2→195,2 | 140 | 20 |
| 13-HpOTrE | 309,2→223,2 | 60 | 6 |
| 10 – HODE | 295,2→183,2 | 140 | 20 |
| 9 – HODE | 295,4→171,2 | 140 | 20 |
| 9-HpOTrE | 309,2→185,2 | 60 | 6 |
| 13 - HPODE | 311,1→113,2 | 80 | 14 |
| 8 – HPODE | 311,1→171,2 | 80 | 14 |
| 9 – oxoODE | 293,2→185,2 | 140 | 18 |
| 11 – HPODE | 311,2→197,2 | 80 | 14 |
| 9 – HPODE | 311,2→185,2 | 80 | 14 |
| 10-HpOME | 313,2→185,2 | 60 | 30 |
| 13 – oxoODE | 293,2→113,2 | 140 | 20 |
| 12(13)-EpOME | 295,2→195,2 | 140 | 14 |
| 9(10)-EpOME | 295,2→171,2 | 140 | 18 |

| Analyte | Transition | Fragmentor(V) | Collision energy (eV) |
| --- | --- | --- | --- |
| PIP 36:2 (18:2; 18:0) | 960,2→339,2 | 140 | 28 |
| PIP 36:2 (18:1; 18:1) | 960,2→337,2 | 140 | 28 |
| PIP 36:3 (18:2; 18:1) | 958,2→337,2 | 140 | 28 |
| PIP 36:4 (18:4; 18:0) | 956,2→339,2 | 140 | 28 |
| PIP 36:4 (18:3;18:1) | 956,2→337,2 | 140 | 28 |
| PIP 36:4 (18:2;18:2) | 956,2→335,2 | 140 | 28 |
| TAG51:0 | 876,4→577,4 | 135 | 28 |
| TAG48:0 | 824,4→551,4 | 130 | 28 |
| PC 34:2 | 758,2→281,2 | 80 | 30 |
| PE 36:3 | 716,4→575,4 | 140 | 26 |
| PE 34:2 | 690,5→549,5 | 140 | 26 |
| OL | 651,5→115,1 | 135 | 28 |
| DAG 36:2 (18:2;18:0) | 638,2→341,2 | 140 | 28 |
| DAG 36:2 (18:1;18:1) | 638,2→339,2 | 140 | 28 |
| DAG 36:3 (18:2;18:1) | 636,2→339,2 | 140 | 28 |
| DAG 36:4 (18:1;18:3) | 634,2→339,2 | 140 | 28 |
| DAG 36:4 (18:2;18:2) | 634,2→337,2 | 140 | 28 |
| DAG34:2 | 610,2→339,2 | 140 | 20 |
| DAG32:2 | 582,2→311,2 | 140 | 20 |
| BHP | 547,5→305,2 | 135 | 24 |
| PIP 36:1 (18:1; 18:0) | 962,2→339,2 | 140 | 28 |
| PG32:1 | 719,2→253,2 | -140 | 28 |
| c23:0 | 353,5→353,5 | -135 | 0 |
| PG34:1 | 747,2→281,2 | -140 | 28 |
| MAG18:0 | 331,2→313,2 | 100 | 18 |
| MAG20:0 | 359,2→341,2 | 100 | 25 |

**B**

**C**

| Analyte | [M-H]- | Fragmentor(V) |
| --- | --- | --- |
| C24:0 | 367,3 | -140 |
| C24:1 | 365,3 | -140 |
| C22:0 | 339,3 | -140 |
| C22:1 | 337,3 | -140 |
| C20:0 | 311,2 | -140 |
| C20:1 | 309,2 | -140 |
| C20:2 | 307,3 | -140 |
| C19:0 | 297,2 | -140 |
| C18:0 | 283,2 | -140 |
| C18:1 | 281,2 | -140 |
| C18:2 | 279,2 | -140 |
| C18:3 | 277,2 | -140 |
| C17:1 | 267,2 | -140 |
| C17:0 | 269,2 | -140 |
| C16:0 | 255,2 | -140 |
| C16:1 | 253,2 | -140 |
| C15:1 | 239,2 | -140 |
| C15:0 | 241,2 | -140 |
| C14:1 cis | 225,2 | -140 |
| C14:0 | 227,2 | -140 |
| C13:0 | 213,2 | -140 |
| C12:0 | 199,2 | -140 |
