# Supplementary material for: Xylella fastidiosa subsp. pauca and olive produced lipids moderate the switch adhesive versus non-adhesive state and viceversa
Source: PLoS One. 2020 May 15;15(5):e0233013. doi: 10.1371/journal.pone.0233013 (PMC7228078; doi:10.1371/journal.pone.0233013)
Supplement: S2 Table — Each row provides the compound putative identification and values explaining differences between Xf-positive and Xf-negative samples, i.e.: p-value, corrected p-value, Fold Change of Xf-positive versus Xf-negative, abundance difference of Xf-positive versus Xf-negative in raw and log2 form. Compounds highlighted in yellow were fragmented and characterized through product ion experiments. (DOCX) [file pone.0233013.s008.docx]

**S2 Table**

| Compound | p | p (Corr) | FC (abs) | Regulation | FC | Abund Diff (Raw) | Abund Diff (Log2) |
| --- | --- | --- | --- | --- | --- | --- | --- |
| DAG 36:4 (18:1;18:3) | 0,005616 | 0,01681 | 2,289478 | up | 2,289478 | -387643 | -18,56437 |
| MAG18:0 | 7,61E-05 | 0,002376 | 3,242852 | up | 3,242852 | 113384,5 | 16,790863 |
| Isomontanic acid | 0,006862 | 0,019473 | 2,154396 | down | -2,1544 | -563039,94 | -19,102879 |
| DAG 36:4 (18:2;18:2) | 0,00242 | 0,010792 | 2,218082 | up | 2,218082 | -80358,25 | -16,294159 |
| Myrrhanol A | 1,00E-04 | 0,00243 | 2,758084 | up | 2,758084 | 300032 | 18,194757 |
| DAG 36:2 (18:2;18:0) | 0,00242 | 0,010792 | 2,232222 | up | 2,232222 | -67545,75 | -16,043577 |
| DAG 36:3 (18:2;18:1) | 0,001551 | 0,008578 | 2,467071 | up | 2,467071 | 75375 | 16,2018 |
| Arachidonic Acid-biotin | 0,001941 | 0,009322 | 2,277424 | up | 2,277424 | 100821 | 16,621437 |
| TAG51:0 | 7,61E-05 | 0,002376 | 3,339292 | up | 3,339292 | 64103 | 15,968104 |
| 4,4'-Diaponeurosporenic acid | 0,010123 | 0,025871 | 2,207658 | up | 2,207658 | -34041,28 | -15,054997 |
| Manalpha1-3Manbeta1-4Glcbeta-Cer(d18:1/16:0) | 0,001234 | 0,007488 | 2,435776 | up | 2,435776 | -15485,531 | -13,918633 |
| 2-hexyl-decanoic acid | 9,78E-04 | 0,006572 | 2,747891 | up | 2,747891 | -24128,688 | -14,558462 |
| (Z)-2-Pentacos-16-enamidoethanesulfonic acid | 0,004578 | 0,015155 | 2,33905 | up | 2,33905 | -18251,75 | -14,155747 |
| C47 H85 N S | 1,01E-02 | 0,025871 | 1,912831 | up | 1,912831 | 4532,5938 | 12,146121 |
| Ganglioside GA2 (d18:1/9Z-18:1) | 0,001234 | 0,007488 | 2,370623 | up | 2,370623 | -22585,406 | -14,463103 |
| 8-Hydroxypurine | 0,014699 | 0,033808 | 2,021973 | up | 2,021973 | -5482,0625 | -12,420503 |
| DAG34:2 | 0,006862 | 0,019473 | 2,075788 | up | 2,075788 | 8263,1875 | 13,012483 |
| DAG32:2 | 0,005616 | 0,01681 | 2,223189 | up | 2,223189 | -17874,125 | -14,125585 |
| PS(14:0/14:1(9Z)) | 6,06E-04 | 0,004731 | 2,438621 | up | 2,438621 | -14607,75 | -13,834446 |
| (6S)-vitamin D2 6,19-sulfur dioxide adduct / (6S)-ergocalciferol 6,19-sulfur dioxide adduct | 0,008351 | 0,02281 | 2,234169 | up | 2,234169 | -12753,25 | -13,638577 |
| Plerixafor | 2,87E-04 | 0,003061 | 2,772983 | up | 2,772983 | 55201,594 | 15,752422 |
| Sitoindoside II | 0,004578 | 0,015155 | 2,362231 | up | 2,362231 | -3361,0312 | -11,714688 |
| MAG20:0 | 7,61E-05 | 0,002376 | 3,087201 | up | 3,087201 | 49297,438 | 15,589225 |
| Leucodelphinidin 3-[galactosyl-(1-4)-glucoside] | 0,005616 | 0,01681 | 2,063256 | up | 2,063256 | 17256,125 | 14,0748205 |
| Isofucosterol 3-O-[6-O-(9-Octadecenoyl)-b-D-glucopyranoside] | 0,004578 | 0,015155 | 2,228999 | up | 2,228999 | -7322,5312 | -12,838127 |
| stigmast-5-en-3beta-ol 3-0-(6'-O-hexadexcanoyl-beta-D-glucopyranoside) | 0,001551 | 0,008578 | 2,259894 | up | 2,259894 | -7862,797 | -12,940826 |
| Trp Met Met | 1,31E-04 | 0,00249 | 1,818291 | up | 1,818291 | 39502,53 | 15,269657 |
| Digeranyl | 0,012223 | 0,030008 | 2,151132 | up | 2,151132 | -15791,781 | -13,946886 |
| DAG 36:3 (18:2;18:1) | 7,61E-05 | 0,002376 | 3,161873 | up | 3,161873 | 13248,656 | 13,693559 |
| N1,N12-Diacetylspermine | 1,71E-04 | 0,00249 | 3,094215 | down | -3,09422 | -257492,83 | -17,974173 |
| DAG 36:2 (18:1;18:1) | 0,010123 | 0,02665 | 2,298462 | up | 2,298462 | 10654,406 | 13,379163 |
| PS(21:0/22:6(4Z,7Z,10Z,13Z,16Z,19Z)) | 0,00242 | 0,010792 | 2,341021 | up | 2,341021 | -9431,297 | -13,20324 |
| Pro Pro Lys - 7.7750626 | 0,001941 | 0,009322 | 2,459926 | down | -2,45993 | -101252,25 | -16,627594 |
| Janthitrem C | 0,017608 | 0,039662 | 2,038058 | up | 2,038058 | -18412,422 | -14,168392 |
| PA(18:0/17:2(9Z,12Z)) | 0,001941 | 0,009322 | 2,273997 | up | 2,273997 | 25844,281 | 14,6575575 |
| 2-Hexaprenyl-3-methyl-6-methoxy-1,4 benzoquinone | 0,003005 | 0,012048 | 2,316221 | up | 2,316221 | -6899,211 | -12,752215 |
| Pheophytin a | 7,71E-04 | 0,005437 | 2,489791 | up | 2,489791 | 12053,359 | 13,557148 |
| 16alpha,17alpha-Epoxy-20-oxopregn-5-en-3beta-yl acetate | 0,005616 | 0,01681 | 2,502817 | up | 2,502817 | 1555,2188 | 10,602901 |
| PIP 36:2 (18:2; 18:0) | 0,003716 | 0,013312 | 2,316647 | up | 2,316647 | -6340,0625 | -12,630281 |
| Glycerol 2-(9Z,12Z-octadecadienoate) 1-hexadecanoate 3-O-[alpha-D-galactopyranosyl-(1-6)-beta-D-galactopyranoside] | 0,017608 | 0,039662 | 2,251703 | up | 2,251703 | 7443,7656 | 12,861817 |
| N1-Acetylthermospermine | 0,008351 | 0,02281 | 2,215291 | up | 2,215291 | -28905,438 | -14,819054 |
| Jubanine C | 0,005616 | 0,01681 | 2,232616 | up | 2,232616 | -6928,125 | -12,758249 |
| N1-Acetylthermospermine - 1.5160046 | 0,012223 | 0,030008 | 2,17361 | up | 2,17361 | -30465,219 | -14,894876 |
| PIP 36:2 (18:1; 18:1) | 0,006862 | 0,019473 | 2,140105 | up | 2,140105 | -4353,047 | -12,08781 |
| PIP 36:3 (18:2; 18:1) | 0,004578 | 0,015155 | 2,28395 | up | 2,28395 | -4718,875 | -12,204227 |
| PIP 36:4 (18:4; 18:0) | 1,31E-04 | 0,00249 | 3,056169 | up | 3,056169 | -13566,5625 | -13,727768 |
| PIP 36:4 (18:3;18:1) | 0,008351 | 0,02281 | 2,102788 | up | 2,102788 | -6987,625 | -12,770586 |
| PIP 36:4 (18:2;18:2) | 7,71E-04 | 0,005437 | 2,795757 | up | 2,795757 | 53601,42 | 15,709984 |
| Nebrosteroid L | 0,012223 | 0,030008 | 2,092259 | up | 2,092259 | -6019,4766 | -12,555423 |
| Trp Met Met - 6.2900467 | 1,71E-04 | 0,00249 | 2,915105 | up | 2,915105 | -20208,281 | -14,302659 |
| PA(13:0/14:0) | 0,006862 | 0,019473 | 2,022111 | up | 2,022111 | -7845,492 | -12,937648 |
| C31 H54 O3 | 7,71E-04 | 0,005437 | 2,565855 | up | 2,565855 | 13568,016 | 13,727922 |
| N1,N12-Diacetylspermine - 9.504081 | 2,22E-04 | 0,002695 | 3,086464 | down | -3,08646 | -255036,36 | -17,960344 |
| Armillatin | 0,003716 | 0,013312 | 2,371896 | down | -2,3719 | -33211,434 | -15,019392 |
| Behenyl alcohol | 0,003716 | 0,013312 | 2,280086 | up | 2,280086 | -6338,9688 | -12,630033 |
| DG(18:0/18:4(6Z,9Z,12Z,15Z)/0:0) | 0,003716 | 0,013312 | 2,351179 | up | 2,351179 | -2317,8984 | -11,178601 |
| PS(22:6(4Z,7Z,10Z,13Z,16Z,19Z)/17:2(9Z,12Z)) | 0,005616 | 0,01681 | 2,262054 | up | 2,262054 | -4268,164 | -12,0594 |
| MG(0:0/16:0/0:0) | 7,61E-05 | 0,002376 | 3,339238 | up | 3,339238 | 8072,0156 | 12,978713 |
| PA(20:4(5Z,8Z,11Z,14Z)/22:6(4Z,7Z,10Z,13Z,16Z,19Z)) | 0,021008 | 0,044567 | 1,850315 | up | 1,850315 | -7094,9688 | -12,792581 |
| 15:0 Cholesteryl ester | 0,014699 | 0,033808 | 1,558579 | up | 1,558579 | -4187,578 | -12,0319 |
| PS(18:0/20:2(11Z,14Z)) | 9,78E-04 | 0,006572 | 1,856886 | up | 1,856886 | 6421,5 | 12,648695 |
| 23-Acetoxysoladulcidine | 0,003005 | 0,012048 | 2,324548 | up | 2,324548 | -3026,7852 | -11,563571 |
| PA(20:1(11Z)/22:6(4Z,7Z,10Z,13Z,16Z,19Z)) | 0,010123 | 0,02665 | 2,169747 | up | 2,169747 | -13782,68 | -13,750568 |
| PI(18:1(9Z)/0:0) | 0,003005 | 0,012048 | 2,34316 | up | 2,34316 | -3417,8594 | -11,738877 |
| N1,N12-Diacetylspermine - 6.3180466 | 3,70E-04 | 0,003592 | 2,657419 | up | 2,657419 | -9215,75 | -13,169886 |
| PS(O-18:0/0:0) | 0,017608 | 0,039662 | 2,039981 | up | 2,039981 | -4929,9297 | -12,267351 |
| TAG51:0 | 1,00E-04 | 0,00243 | 3,123453 | up | 3,123453 | 22535,328 | 14,459901 |
| TAG48:0 | 4,75E-04 | 0,004066 | 2,676542 | up | 2,676542 | -7487,367 | -12,870243 |
| PC 34:2 | 7,61E-05 | 0,002376 | 3,162173 | up | 3,162173 | 17626,828 | 14,105485 |
| Phylloquinone | 7,61E-05 | 0,002376 | 3,301449 | up | 3,301449 | 835,59766 | 9,706665 |
| Kynostatin 272 | 0,004578 | 0,015155 | 2,32947 | up | 2,32947 | -2690,1484 | -11,39347 |
| PE 36:3 | 1,31E-04 | 0,00249 | 3,164765 | up | 3,164765 | 2296,3203 | 11,165108 |
| PE 34:2 | 0,003716 | 0,013312 | 2,367641 | up | 2,367641 | -649,7539 | -9,34375 |
| Thalicsessine | 6,06E-04 | 0,004731 | 2,576181 | up | 2,576181 | 8002,9688 | 12,966319 |
| PS(22:6(4Z,7Z,10Z,13Z,16Z,19Z)/0:0) | 0,003716 | 0,013312 | 2,254398 | up | 2,254398 | -2500,0117 | -11,287719 |
| SQDG(16:0/16:0) | 1,71E-04 | 0,00249 | 2,85647 | up | 2,85647 | 4090,4805 | 11,9980545 |
| PA(22:6(4Z,7Z,10Z,13Z,16Z,19Z)/15:1(9Z)) | 0,00242 | 0,010792 | 2,482218 | up | 2,482218 | -5372,4688 | -12,39137 |
| PE-Cer(d16:2(4E,6E)/18:1(9Z)) | 0,003716 | 0,013312 | 2,369935 | down | -2,36993 | -17650,465 | -14,107419 |
| 21-Methylhentriacontan-7-one | 0,001941 | 0,009322 | 2,443189 | up | 2,443189 | 4642,7773 | 12,180773 |
| PIP 36:1 (18:1; 18:0) | 0,001941 | 0,009322 | 2,03092 | up | 2,03092 | 4734,1836 | 12,2088995 |
| PG32:1 | 0,005616 | 0,01681 | 2,231779 | down | -2,23178 | -43178,727 | -15,398033 |
| PS(14:1(9Z)/14:1(9Z)) | 0,003716 | 0,013312 | 2,231958 | up | 2,231958 | -886,2656 | -9,791595 |
| Dihydrospheroidene/ Methoxyneurosporene | 2,87E-04 | 0,003061 | 2,783685 | up | 2,783685 | 17795,305 | 14,119209 |
| NeuAcalpha2-3Galbeta-Cer(d18:1/24:0) | 2,22E-04 | 0,002695 | 2,941831 | down | -2,94183 | -22596,852 | -14,463834 |
| Met Arg Met | 0,005616 | 0,01681 | 2,352548 | down | -2,35255 | -33107,58 | -15,0148735 |
| PG(O-16:0/14:0) | 0,005616 | 0,01681 | 1,62394 | up | 1,62394 | -2464,293 | -11,266958 |
| Deterrol stearate | 0,001551 | 0,008578 | 2,613119 | up | 2,613119 | 3017,6035 | 11,559188 |
| Arg Trp Val | 0,021008 | 0,044567 | 2,183055 | up | 2,183055 | -1620,1758 | -10,661935 |
| PG34:1 | 0,006862 | 0,019473 | 2,03941 | up | 2,03941 | -2504,3438 | -11,290216 |
| 2-Octaprenyl-6-hydroxyphenol | 0,021008 | 0,044567 | 1,987072 | up | 1,987072 | -2535,4453 | -11,308023 |
| PI(20:3(8Z,11Z,14Z)/15:0) - 14.320105 | 1,00E-04 | 0,00243 | 2,835496 | up | 2,835496 | 4239,199 | 12,049576 |
| MGDG(16:0/18:2(9Z,12Z)) | 2,22E-04 | 0,002695 | 2,730612 | up | 2,730612 | 3544,496 | 11,791365 |
| 1α-hydroxy-23-[3-(1-hydroxy-1-methylethyl)phenyl]-22,22,23,23-tetradehydro-24,25,26,27-tetranorvitamin D3 / 1α-hydroxy-23-[3-(1-hydroxy-1-methylethyl)phenyl]-22,22,23,23-tetradehydro-24,25,26,27-tetranorcholecalciferol | 9,78E-04 | 0,006572 | 2,461751 | up | 2,461751 | -1601,0234 | -10,644778 |
| 3-Tritriacontanone | 0,001234 | 0,007488 | 2,510537 | up | 2,510537 | 8665,426 | 13,081055 |
| Cer(d18:2/21:0) | 4,75E-04 | 0,004066 | 2,690288 | up | 2,690288 | 379,89844 | 8,56947 |
| 1-O-alpha-D-glucopyranosyl-(2-tetradecanoyloxy)-eicosan-1-ol | 0,003005 | 0,012048 | 2,114119 | up | 2,114119 | 3015,4648 | 11,558165 |
| 3-O-(2-O-(2E-decenoyl)-alpha-L-rhamnopyranosyl-(1-2)-alpha-L-rhamnopyranosyl)-3-hydroxydecanoic acid | 0,001551 | 0,008578 | 2,588402 | up | 2,588402 | 5340,754 | 12,382828 |
| Z-Arg-Arg-NHMec | 2,87E-04 | 0,003061 | 2,339008 | up | 2,339008 | -1862,2891 | -10,862862 |
| Crasseride 2a | 7,61E-05 | 0,002376 | 3,102162 | up | 3,102162 | 5090,0723 | 12,313471 |
| Ser Trp Met | 3,70E-04 | 0,003592 | 2,696529 | down | -2,69653 | -42523,766 | -15,375981 |
| Ser Trp Met - 9.49308 | 2,22E-04 | 0,002695 | 2,833737 | down | -2,83374 | -44072,445 | -15,427589 |
| Methyl Gamboginate | 0,00242 | 0,010792 | 2,162667 | up | 2,162667 | -1544,9824 | -10,593374 |
| Glycerol 1,3-di-(9Z,12Z-octadecadienoate) 2-octadecanoate | 0,001941 | 0,009322 | 2,371757 | up | 2,371757 | -2380,627 | -11,217126 |
| Met Thr Trp | 0,00242 | 0,010792 | 2,337762 | up | 2,337762 | 183,32617 | 7,518269 |
| TG(17:2(9Z,12Z)/22:2(13Z,16Z)/22:4(7Z,10Z,13Z,16Z))[iso6] | 0,001234 | 0,007488 | 2,569481 | down | -2,56948 | -12439,157 | -13,602601 |
| 2-Hexaprenyl-3-methyl-5-hydroxy-6-methoxy-1,4-benzoquinol | 0,014699 | 0,033808 | 2,119389 | up | 2,119389 | -2235,6914 | -11,126505 |
| NAPE(16:0/18:1(9Z)/20:4(5Z,8Z,11Z,14Z) | 0,014699 | 0,033808 | 2,256681 | up | 2,256681 | 3067,6387 | 11,582912 |
| PE-Cer(d14:2(4E,6E)/16:0) | 0,012223 | 0,030008 | 1,677383 | up | 1,677383 | -1609,4023 | -10,652309 |
| Manalpha1-3Manbeta1-4Glcbeta-Cer(d18:1/24:0) | 0,004578 | 0,015155 | 2,187435 | up | 2,187435 | -2357,879 | -11,203274 |
| FMC-5(d18:1/24:1) | 2,87E-04 | 0,003061 | 2,605813 | up | 2,605813 | -612,209 | -9,25788 |
| (+)-24-Methyl-hexacosanoic acid - 18.691067 | 4,75E-04 | 0,004066 | 2,892709 | up | 2,892709 | 3196,891 | 11,642454 |
| N-Desethylquinagolide | 0,010123 | 0,02665 | 2,066183 | up | 2,066183 | 2110,0781 | 11,04308 |
| iso-1,2-octadecanediol | 7,61E-05 | 0,002376 | 3,338441 | up | 3,338441 | 8505,234 | 13,054135 |
| 18:2-Glc-Sitosterol | 0,001234 | 0,007488 | 2,286686 | up | 2,286686 | -4912,9688 | -12,26238 |
| Arg Met Val | 0,003005 | 0,012048 | 2,558635 | up | 2,558635 | -1059,0078 | -10,048497 |
| Arachidonoyl Serotonin | 0,003716 | 0,013312 | 2,242298 | up | 2,242298 | 235,11523 | 7,8772244 |
| PE(16:0/18:1(9Z))-15-isoLG pyrrole | 0,005616 | 0,01681 | 2,166027 | up | 2,166027 | -2345,9023 | -11,195928 |
| Leucodelphinidin 3-[galactosyl-(1-4)-glucoside] - 7.3050566 | 0,001941 | 0,009322 | 2,226397 | up | 2,226397 | 4082,8398 | 11,9953575 |
| 1-(O-alpha-D-glucopyranosyl)-(1,3R,25S,27R)-octacosanetetraol | 0,005616 | 0,01681 | 2,279377 | up | 2,279377 | -2283,8281 | -11,157238 |
| Arg Asn Arg | 0,012223 | 0,030008 | 2,327209 | up | 2,327209 | 1145,8809 | 10,162241 |
| 1-Hydroxyepiacorone | 0,012223 | 0,030008 | 1,955264 | up | 1,955264 | -791,6836 | -9,62878 |
| PG(20:0/17:2(9Z,12Z)) | 7,71E-04 | 0,005437 | 2,769721 | up | 2,769721 | 2249,8848 | 11,135635 |
| Manzamine A | 0,003005 | 0,012048 | 2,308448 | up | 2,308448 | -129,81445 | -7,020307 |
| Latanoprost ethyl amide | 1,31E-04 | 0,00249 | 2,547063 | up | 2,547063 | -766,2344 | -9,581642 |
| bis(7)-Tacrine | 0,004578 | 0,015155 | 2,154929 | up | 2,154929 | 5153,0703 | 12,331217 |
| (±)12,13-DiHOME-d4 | 0,006862 | 0,019473 | 2,335271 | up | 2,335271 | -1881,5527 | -10,877708 |
| TG(12:0/12:0/20:4(5Z,8Z,11Z,14Z))[iso3] | 7,61E-05 | 0,002376 | 3,454755 | up | 3,454755 | 2450,0254 | 11,258581 |
| N-Desethylquinagolide - 9.51008 | 1,71E-04 | 0,00249 | 3,030133 | down | -3,03013 | -81520,18 | -16,314869 |
| beta-D-Glucosyloxydestruxin B | 0,005616 | 0,01681 | 2,212277 | up | 2,212277 | -787,1504 | -9,620496 |
| Manalpha1-3Manbeta1-4Glcbeta-Cer(d18:1/16:0) - 19.786041 | 0,001234 | 0,007488 | 2,382591 | up | 2,382591 | -864,66797 | -9,756002 |
| PS(17:1(9Z)/19:1(9Z)) | 0,006862 | 0,019473 | 2,148031 | up | 2,148031 | -865,41504 | -9,757248 |
| Latanoprost ethyl amide - 11.727099 | 1,31E-04 | 0,00249 | 2,552706 | up | 2,552706 | -746,14844 | -9,543319 |
| Elastin | 0,021008 | 0,044567 | 2,039427 | up | 2,039427 | -864,80664 | -9,756234 |
| (+)-24-Methyl-hexacosanoic acid - 18.691067 :6 | 4,75E-04 | 0,004066 | 2,91401 | up | 2,91401 | 3272,919 | 11,676362 |
| Arachidonyl Trifluoromethyl Ketone | 0,001941 | 0,009322 | 2,035952 | up | 2,035952 | 2437,5527 | 11,251218 |
| 1-(10-methyl-hexadecanoyl-2-(8-[3]-ladderane-octanyl)-sn-glycerol | 3,70E-04 | 0,003592 | 2,75012 | up | 2,75012 | 4199,7754 | 12,036097 |
| TG(22:4(7Z,10Z,13Z,16Z)/22:4(7Z,10Z,13Z,16Z)/22:6(4Z,7Z,10Z,13Z,16Z,19Z))[iso3] | 0,010123 | 0,02665 | 2,145404 | up | 2,145404 | 1489,8281 | 10,54093 |
| 2-Methoxyestradiol-17β 3-sulfate | 0,008351 | 0,02281 | 2,026756 | up | 2,026756 | 553,24414 | 9,111773 |
| C59 H108 N O9 P | 0,004578 | 0,015155 | 2,410829 | up | 2,410829 | 3092,1875 | 11,594412 |
| 17beta-Hydroxy-4-mercaptoandrost-4-en-3-one 4-acetate 17-propionate | 1,71E-04 | 0,00249 | 3,030976 | up | 3,030976 | 2621,336 | 11,356087 |
| Arg Val Cys | 0,001941 | 0,009322 | 2,40514 | up | 2,40514 | 108,56641 | 6,762434 |
| (6S)-vitamin D2 6,19-sulfur dioxide adduct / (6S)-ergocalciferol 6,19-sulfur dioxide adduct - 12.127102 | 0,003005 | 0,012048 | 2,235091 | up | 2,235091 | -11214,031 | -13,453017 |
| Campesteryl caffeate | 0,021008 | 0,044567 | 1,708304 | up | 1,708304 | -102,58203 | -6,680634 |
| PA(19:1(9Z)/22:4(7Z,10Z,13Z,16Z)) | 0,017608 | 0,039662 | 2,04799 | up | 2,04799 | -100,23828 | -6,6472898 |
| Araliacerebroside | 0,001941 | 0,009322 | 2,406291 | down | -2,40629 | -13890,848 | -13,761847 |
| 17beta-Hydroxy-4-mercaptoandrost-4-en-3-one 4-acetate 17-propionate - 6.298045 | 1,71E-04 | 0,00249 | 3,012182 | up | 3,012182 | 2429,543 | 11,2464695 |
| TG(16:1(9Z)/22:2(13Z,16Z)/22:2(13Z,16Z))[iso3] | 0,008351 | 0,02281 | 2,142341 | up | 2,142341 | -1459,1562 | -10,510919 |
| (3S,3'S,5R,5'R,6R)-6,7-Didehydro-5,6-dihydro-3,3',5,8'-tetrahydroxy-beta,kappa-caroten-6'-one | 0,021008 | 0,044567 | 1,994121 | up | 1,994121 | -4893,2344 | -12,256573 |
| Niddamycin | 0,014699 | 0,033808 | 1,996771 | up | 1,996771 | -910,03125 | -9,829772 |
| Pyrohyperforin | 0,001941 | 0,009322 | 2,440093 | up | 2,440093 | -986,7822 | -9,946588 |
| 6,8a-Seco-6,8a-deoxy-5-oxoavermectin ''2a'' aglycone | 0,005616 | 0,01681 | 2,254741 | up | 2,254741 | -1972,7539 | -10,945995 |
| Mycolactone | 0,005616 | 0,01681 | 2,236542 | down | -2,23654 | -8370,084 | -13,031027 |
| Phe Lys Tyr | 0,021008 | 0,044567 | 2,121222 | up | 2,121222 | -611,43945 | -9,256065 |
| Neoacrimarine H | 0,021008 | 0,044567 | 1,95785 | up | 1,95785 | 2273,7656 | 11,150867 |
| 1α,25-dihydroxy-11-(4-hydroxymethylphenyl)-9,11-didehydrovitamin D3 / 1α,25-dihydroxy-11-(4-hydroxymethylphenyl)-9,11-didehydrocholecalciferol - 12.571103 | 0,001551 | 0,008578 | 2,326149 | up | 2,326149 | -523,0371 | -9,030769 |
| EB 1213 | 6,06E-04 | 0,004731 | 2,687422 | up | 2,687422 | 193,45703 | 7,5958695 |
| Bisabolol oxide A | 0,003005 | 0,012048 | 2,19929 | up | 2,19929 | -329,86426 | -8,365728 |
| Cavipetin D | 0,014699 | 0,033808 | 2,146449 | up | 2,146449 | -503,2754 | -8,975204 |
| Sativic acid | 7,61E-05 | 0,002376 | 3,321971 | up | 3,321971 | 907,68945 | 9,826055 |
| Met Thr Arg | 0,010123 | 0,02665 | 2,089864 | up | 2,089864 | -1651,1699 | -10,689273 |
| TG(20:5(5Z,8Z,11Z,14Z,17Z)/22:2(13Z,16Z)/22:6(4Z,7Z,10Z,13Z,16Z,19Z))[iso6] | 0,003716 | 0,013312 | 2,440648 | up | 2,440648 | 1447,04 | 10,498889 |
| PE-Cer(d16:1(4E)/21:0) | 0,012223 | 0,030008 | 1,999763 | up | 1,999763 | 2258,795 | 11,141337 |
| Sativic acid - 3.0950148 | 7,61E-05 | 0,002376 | 3,317423 | up | 3,317423 | 920,5176 | 9,846301 |
| Neoacrimarine H - 6.462047 | 0,021008 | 0,044567 | 1,95785 | up | 1,95785 | 2273,7656 | 11,150867 |
| Avermectin A2a | 0,003716 | 0,013312 | 2,545358 | up | 2,545358 | 1146,0029 | 10,1623955 |
| Heneicosanol | 2,87E-04 | 0,003061 | 2,828767 | up | 2,828767 | 3278,1016 | 11,678645 |
| Araliasaponin II | 0,021008 | 0,044567 | 1,986248 | up | 1,986248 | -1628,999 | -10,66977 |
| PE-Cer(d16:1(4E)/22:0) | 0,010123 | 0,025871 | 2,092872 | up | 2,092872 | -212,85938 | -7,733757 |
| Janthitrem C - 14.622104 | 0,014699 | 0,033808 | 2,034639 | up | 2,034639 | -18455,078 | -14,17173 |
| PE(22:6(4Z,7Z,10Z,13Z,16Z,19Z)/21:0) | 0,021008 | 0,044567 | 2,044908 | up | 2,044908 | -181,04297 | -7,5001884 |
| PA(18:3(6Z,9Z,12Z)/15:1(9Z)) | 0,001551 | 0,008578 | 2,36403 | up | 2,36403 | -496,43848 | -8,955471 |
| 2-decaprenyl-6-methoxy-3-methyl-1,4-benzoquinone | 2,22E-04 | 0,002695 | 2,851111 | up | 2,851111 | 2658,228 | 11,376249 |
| PA(17:1(9Z)/22:0) | 0,003716 | 0,013312 | 1,63135 | up | 1,63135 | 16355,9375 | 13,997527 |
| Feruloyldihydro-beta-sitosterol | 1,00E-04 | 0,00243 | 3,000325 | up | 3,000325 | 3900,293 | 11,929367 |
| Sorbitan palmitate | 0,014699 | 0,033808 | 2,439595 | up | 2,439595 | -774,15234 | -9,596474 |
| PA(15:1(9Z)/15:1(9Z)) | 1,71E-04 | 0,00249 | 2,947055 | up | 2,947055 | 11587,516 | 13,500283 |
| Natamycin | 0,010123 | 0,025871 | 2,243382 | up | 2,243382 | -584,5244 | -9,191119 |
| ergosteryl palmitoleate | 7,61E-05 | 0,002376 | 3,226243 | up | 3,226243 | 1154,6855 | 10,173285 |
| 12-Tricosanol | 6,06E-04 | 0,004731 | 3,033474 | up | 3,033474 | 4014,6416 | 11,971055 |
| Polyoxyethylene 40 monostearate | 4,75E-04 | 0,004066 | 2,74941 | up | 2,74941 | 3065,0488 | 11,581695 |
| TG(15:0/22:3(10Z,13Z,16Z)/22:3(10Z,13Z,16Z))[iso3] | 0,003005 | 0,012048 | 2,279245 | down | -2,27925 | -3933,958 | -11,941766 |
| PA(15:0/12:0) | 0,021008 | 0,044567 | 2,019959 | up | 2,019959 | -7203,6016 | -12,814503 |
| PI(O-20:0/17:0) | 7,71E-04 | 0,005437 | 2,813047 | up | 2,813047 | 128,08203 | 7,000924 |
| Feruloyldihydro-beta-sitosterol - 13.335108 | 2,22E-04 | 0,002695 | 3,006212 | up | 3,006212 | 2831,209 | 11,467202 |
| Sorbitan palmitate - 6.886052 | 0,014699 | 0,033808 | 2,432566 | up | 2,432566 | -914,3711 | -9,836636 |
| 1-(O-alpha-D-glucopyranosyl)-3-keto-(1,25R)-hexacosanediol | 0,008351 | 0,02281 | 2,090103 | up | 2,090103 | -315,50195 | -8,301505 |
| alpha-L-Rhamnopyranosyl-(1-3)-alpha-D-galactopyranosyl-(1-3)-L-fucose | 0,001551 | 0,008578 | 2,353476 | up | 2,353476 | 580,6172 | 9,181443 |
| Ganglioside GM3 (d18:1/20:0) | 0,004578 | 0,015155 | 2,43757 | up | 2,43757 | 1232,708 | 10,267615 |
| PS(22:6(4Z,7Z,10Z,13Z,16Z,19Z)/20:1(11Z)) | 0,003005 | 0,012048 | 2,345008 | up | 2,345008 | -177,11523 | -7,4685445 |
| Janthitrem E | 0,003005 | 0,012048 | 2,361224 | up | 2,361224 | -239,9336 | -7,9064913 |
| (25R)-3alpha,7alpha-dihydroxy-5beta-cholestan-27-oyl taurine | 0,014699 | 0,033808 | 2,033335 | up | 2,033335 | 3484,4102 | 11,766699 |
| 13-beta-D-Glucosyloxydocosanoate | 0,004578 | 0,015155 | 2,148196 | up | 2,148196 | 853,1201 | 9,736605 |
| PA(12:0/20:3(8Z,11Z,14Z)) - 18.533066 | 3,70E-04 | 0,003592 | 2,740805 | up | 2,740805 | 1618,2715 | 10,660238 |
| KN-62 | 0,003716 | 0,013312 | 2,342461 | up | 2,342461 | -141,01953 | -7,139751 |
| 3-Tritriacontanone - 17.439085 | 0,001234 | 0,007488 | 2,509949 | up | 2,509949 | 8677,988 | 13,083145 |
| 2-methylbacteriohopane-32,33,34,35-tetrol | 7,61E-05 | 0,002376 | 2,94195 | up | 2,94195 | 5696,965 | 12,475978 |
| Madlongiside D | 7,71E-04 | 0,005437 | 2,444913 | up | 2,444913 | 59,148926 | 5,88628 |
| NeuAcalpha2-3Galbeta-Cer(d18:1/22:0) | 0,014699 | 0,033808 | 2,009999 | up | 2,009999 | 261,83594 | 8,032519 |
| 3-O-Protocatechuoylceanothic acid | 0,00242 | 0,010792 | 1,975597 | up | 1,975597 | -46,799805 | -5,5484304 |
| Feruloyl-beta-sitosterol | 0,014699 | 0,033808 | 1,948575 | up | 1,948575 | -328,37012 | -8,3591795 |
| TG(22:5(7Z,10Z,13Z,16Z,19Z)/22:5(7Z,10Z,13Z,16Z,19Z)/22:5(7Z,10Z,13Z,16Z,19Z)) | 4,75E-04 | 0,004066 | 2,589501 | up | 2,589501 | 1211,3267 | 10,2423725 |
| TG(20:2(11Z,14Z)/22:6(4Z,7Z,10Z,13Z,16Z,19Z)/22:6(4Z,7Z,10Z,13Z,16Z,19Z))[iso3] | 0,001941 | 0,009322 | 2,3045 | up | 2,3045 | 725,7407 | 9,50331 |
| 1,25-Dihydroxy-20S-21-(3-hydroxy-3-methylbutyl)-23-yne-26,27-hexafluorovitamin D3 | 0,010123 | 0,02665 | 2,074956 | up | 2,074956 | 4948,6875 | 12,27283 |
| Octacosanoic acid | 0,006862 | 0,019473 | 2,283965 | down | -2,28397 | -6682,204 | -12,706108 |
| PG(P-16:0/0:0) | 6,06E-04 | 0,004731 | 2,732802 | up | 2,732802 | 3993,711 | 11,963514 |
