# Supplementary material for: Xylella fastidiosa subsp. pauca and olive produced lipids moderate the switch adhesive versus non-adhesive state and viceversa
Source: PLoS One. 2020 May 15;15(5):e0233013. doi: 10.1371/journal.pone.0233013 (PMC7228078; doi:10.1371/journal.pone.0233013)
Supplement: S3 Table — Compounds’ peak areas were divided by their internal standard’s peak area and then by the maximum value of internal standard peak area. For each compound the fold-change of Xf+ samples versus Xf- samples was calculated, as such the p-value from Student T-test. Compounds with a p-value <0.05 are represented on the upper part of the volcano plot, above the red line corresponding to -log10(0.05). (DOCX) [file pone.0233013.s009.docx]

S3 Table

|  |  | Relative abundances | | | | | | | | | | | | | | | | | | | | | | | | |
| --- | --- | --- | --- | --- | --- | --- | --- | --- | --- | --- | --- | --- | --- | --- | --- | --- | --- | --- | --- | --- | --- | --- | --- | --- | --- | --- |
| pool | Xf-positive or negative | 10-HODE Results | 10-HpOME Results | 13-HODE Results | 13-HOTrE Results | 13-oxoODE Results | 8-HODE Results | 9,10 di HOME Results | 9-HODE Results | 9-HOTrE Results | MAG18:0 Results | MAG20:0 Results | BHP Results | DAG 36:3 (18:2;18:1) Results | DAG 36:4 (18:1;18:3) Results | DAG 36:4 (18:2;18:2) Results | DAG34:2 Results | PC 34:2 Results | C12:0 Results | C14:0 Results | C16:0 Results | C16:1 Results | C18:0 Results | C18:1 Results | C18:2 Results | C18:3 Results |
| pool1 | Xf-negative | 921,506 | 3228,669 | 1825,629 | 309,6355 | 229,1475 | 527,6579 | 300,7654 | 678,4062 | 765,527 | 153942,6 | 14130,8 | 25185,39 | 1789,457 | 913,4452 | 8177,258 | 3113,418 | 37692,85 | 2441,396 | 408223,5 | 255628,6 | 9167,1 | 343848,3 | 37057,71 | 36191,86 | 19940,12 |
| pool2 | Xf-negative | 668,5545 | 2345,18 | 1590,434 | 423,7844 | 174,097 | 213,7597 | 258,5583 | 528,2806 | 929,404 | 145806,9 | 12316,23 | 23478,15 | 1499,412 | 798,6292 | 8404,115 | 2451,018 | 51295,36 | 2983,17 | 412683,7 | 233744 | 5519,78 | 243319,5 | 30498 | 22406,02 | 21450,15 |
| pool3 | Xf-negative | 942,8257 | 2722,199 | 1580,191 | 312,6364 | 166,1115 | 185,6194 | 189,8687 | 444,9766 | 438,3302 | 109452,1 | 8855,644 | 21932,99 | 535,4674 | 966,3763 | 3665,041 | 7854,948 | 146227,9 | 1579,804 | 281143,2 | 168201,9 | 5391,866 | 167884,8 | 25716,62 | 21457,56 | 14707,4 |
| pool4 | Xf-negative | 805,2286 | 2420,54 | 1618,732 | 354,1959 | 149,1071 | 214,7204 | 192,3843 | 480,3284 | 542,2969 | 145434,2 | 12407,07 | 18715,02 | 1108,622 | 1167,375 | 7623,972 | 10388,38 | 509037,9 | 1558,078 | 342769,3 | 195912,9 | 5378,908 | 183286,5 | 28757,69 | 23348,95 | 20920,99 |
| pool5 | Xf-negative | 1579,092 | 6374,268 | 2790,774 | 776,4339 | 373,7886 | 567,8544 | 208,6229 | 805,2228 | 1329,774 | 104626,3 | 9271,094 | 20762,37 | 611,93 | 1175,658 | 2949,185 | 4583,195 | 159244,1 | 1421,406 | 274466,9 | 171239,6 | 5992,826 | 159112,8 | 27944,7 | 25766,08 | 17804,76 |
| pool6 | Xf-negative | 688,8282 | 3104,369 | 1649,572 | 396,4997 | 150,4546 | 337,9242 | 207,0457 | 472,6786 | 729,1198 | 97648,94 | 8143,657 | 15365,86 | 850,969 | 874,1038 | 4815,895 | 14302,74 | 464961,8 | 1289,245 | 294140,2 | 166916,4 | 4708,735 | 196584,3 | 25805,46 | 20385,72 | 15340,57 |
| pool7 | Xf-positive | 1607,804 | 4957,684 | 3524,991 | 538,0775 | 239,0933 | 394,6278 | 236,8256 | 1038,16 | 1822,028 | 99411,75 | 8215,372 | 19654,19 | 765,9295 | 1814,566 | 12705,08 | 1125,39 | 24198,01 | 4133,83 | 289423,1 | 179752,8 | 9779,859 | 195507,5 | 41651,29 | 45259,24 | 21740,41 |
| pool8 | Xf-positive | 2834,126 | 4916,992 | 2365,724 | 650,9615 | 254,5252 | 487,0806 | 244,3798 | 664,0568 | 1103,226 | 138175,6 | 11365,43 | 25183,4 | 1052,238 | 3762,728 | 7418,062 | 5674,888 | 325267,2 | 1436,69 | 228978,7 | 152175,9 | 5742,777 | 167753,1 | 37316,74 | 50052,8 | 17979,45 |
| pool9 | Xf-positive | 1187,307 | 4830,915 | 2775,026 | 455,8009 | 300,5789 | 482,0125 | 264,576 | 973,0651 | 1140,992 | 118607,8 | 9835,529 | 20837,56 | 1251,974 | 1890,781 | 7213,002 | 5518,431 | 99961,86 | 1519,487 | 347742,5 | 206499,2 | 6470,892 | 226032,9 | 31661,67 | 53699,23 | 18243,55 |
| pool10 | Xf-positive | 2291,438 | 4001,269 | 2604,543 | 458,0348 | 232,9852 | 354,6857 | 210,7045 | 789,3737 | 1595,839 | 115429,1 | 9500,058 | 22973,59 | 914,6242 | 2299,455 | 12269,73 | 2701,484 | 29919,16 | 1172,706 | 270502,6 | 164608,5 | 6753,519 | 167045,1 | 45161,41 | 56923,03 | 22101,42 |
| pool11 | Xf-positive | 1903,197 | 6239,248 | 4149,047 | 723,0455 | 508,3534 | 505,261 | 271,9074 | 1258,844 | 1192,32 | 158222,7 | 12846,63 | 13613,26 | 630,2441 | 2803,171 | 5463,995 | 5246,18 | 131621,6 | 1904,97 | 384209,3 | 233758,3 | 8928,392 | 288701,5 | 40180,05 | 69607,35 | 28209,91 |
| pool12 | Xf-positive | 1549,289 | 4138,94 | 2974,715 | 529,0035 | 491,362 | 333,198 | 161,018 | 1087,958 | 1286,97 | 90023,3 | 6919,582 | 13660,56 | 910,6819 | 572,4267 | 4381,872 | 11425,07 | 534320,5 | 3363,763 | 399407,2 | 221708,8 | 8104,667 | 306407,3 | 47535,6 | 64790,75 | 24056,34 |
|  |  |  |  |  |  |  |  |  |  |  |  |  |  |  |  |  |  |  |  |  |  |  |  |  |  |  |
|  |  |  |  |  |  |  |  |  |  |  |  |  |  |  |  |  |  |  |  |  |  |  |  |  |  |  |
|  |  | Student T Test (Xf-positive vs Xf-negative) | | | | | | | | | | | | | | | | | | | | | | | | |
|  |  | 10-HODE | 10-HpOME | 13-HODE | 13HOTrE | 13-oxoODE | 8-HODE | 9,10 di HOME | 9-HODE | 9-HOTrE | MAG18:0 | MAG20:0 | BHP | DAG 36:3 (18:2;18:1) | DAG 36:4 (18:1;18:3) | DAG 36:4 (18:2;18:2) | DAG34:2 | PC 34:2 | C12:0 | C14:0 | C16:0 | C16:1 | C18:0 | C18:1 | C18:2 | C18:3 |
|  | p value | 0,005478 | 0,000602 | 0,003042 | 0,004357 | 0,024359 | 0,116157 | 0,947849 | 0,002324 | 0,001365 | 0,508666 | 0,361659 | 0,553837 | 0,369724 | 0,035114 | 0,34819 | 0,40667 | 0,707022 | 0,639683 | 0,495257 | 0,633305 | 0,155675 | 0,966508 | 0,006326 | 8,90E-05 | 0,12501 |
|  |  |  |  |  |  |  |  |  |  |  |  |  |  |  |  |  |  |  |  |  |  |  |  |  |  |  |
|  |  |  |  |  |  |  |  |  |  |  |  |  |  |  |  |  |  |  |  |  |  |  |  |  |  |  |
|  | fold change | 2,028735 | 1,440194 | 1,663817 | 1,303802 | 1,631036 | 1,248752 | 1,0237 | 1,704293 | 1,719602 | 0,951063 | 0,901083 | 0,924129 | 0,863948 | 2,229316 | 1,387711 | 0,742298 | 0,836918 | 1,200331 | 0,953729 | 0,97219 | 1,26607 | 1,044366 | 1,385291 | 2,275616 | 1,201219 |
|  |  |  |  |  |  |  |  |  |  |  |  |  |  |  |  |  |  |  |  |  |  |  |  |  |  |  |
| 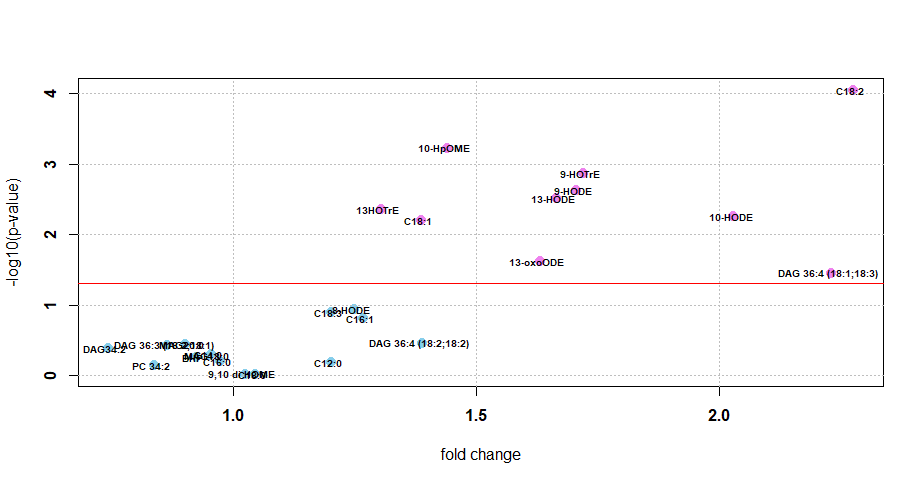   \|  \| \| --- \| |  |  |  |  |  |  |  |  |  |  |  |  |  |  |  |  |  |  |  |  |  |  |  |  |  |  |
|  |  |  |  |  |  |  |  |  |  |  |  |  |  |  |  |  |  |  |  |  |  |  |  |  |  |  |
|  |  |  |  |  |  |  |  |  |  |  |  |  |  |  |  |  |  |  |  |  |  |  |  |  |  |  |
|  |  |  |  |  |  |  |  |  |  |  |  |  |  |  |  |  |  |  |  |  |  |  |  |  |  |  |
|  |  |  |  |  |  |  |  |  |  |  |  |  |  |  |  |  |  |  |  |  |  |  |  |  |  |  |
|  |  |  |  |  |  |  |  |  |  |  |  |  |  |  |  |  |  |  |  |  |  |  |  |  |  |  |
|  |  |  |  |  |  |  |  |  |  |  |  |  |  |  |  |  |  |  |  |  |  |  |  |  |  |  |
|  |  |  |  |  |  |  |  |  |  |  |  |  |  |  |  |  |  |  |  |  |  |  |  |  |  |  |
|  |  |  |  |  |  |  |  |  |  |  |  |  |  |  |  |  |  |  |  |  |  |  |  |  |  |  |
|  |  |  |  |  |  |  |  |  |  |  |  |  |  |  |  |  |  |  |  |  |  |  |  |  |  |  |
|  |  |  |  |  |  |  |  |  |  |  |  |  |  |  |  |  |  |  |  |  |  |  |  |  |  |  |
|  |  |  |  |  |  |  |  |  |  |  |  |  |  |  |  |  |  |  |  |  |  |  |  |  |  |  |
|  |  |  |  |  |  |  |  |  |  |  |  |  |  |  |  |  |  |  |  |  |  |  |  |  |  |  |
|  |  |  |  |  |  |  |  |  |  |  |  |  |  |  |  |  |  |  |  |  |  |  |  |  |  |  |
|  |  |  |  |  |  |  |  |  |  |  |  |  |  |  |  |  |  |  |  |  |  |  |  |  |  |  |
|  |  |  |  |  |  |  |  |  |  |  |  |  |  |  |  |  |  |  |  |  |  |  |  |  |  |  |
|  |  |  |  |  |  |  |  |  |  |  |  |  |  |  |  |  |  |  |  |  |  |  |  |  |  |  |
|  |  |  |  |  |  |  |  |  |  |  |  |  |  |  |  |  |  |  |  |  |  |  |  |  |  |  |
|  |  |  |  |  |  |  |  |  |  |  |  |  |  |  |  |  |  |  |  |  |  |  |  |  |  |  |
|  |  |  |  |  |  |  |  |  |  |  |  |  |  |  |  |  |  |  |  |  |  |  |  |  |  |  |
|  |  |  |  |  |  |  |  |  |  |  |  |  |  |  |  |  |  |  |  |  |  |  |  |  |  |  |
|  |  |  |  |  |  |  |  |  |  |  |  |  |  |  |  |  |  |  |  |  |  |  |  |  |  |  |
|  |  |  |  |  |  |  |  |  |  |  |  |  |  |  |  |  |  |  |  |  |  |  |  |  |  |  |
|  |  |  |  |  |  |  |  |  |  |  |  |  |  |  |  |  |  |  |  |  |  |  |  |  |  |  |
|  |  |  |  |  |  |  |  |  |  |  |  |  |  |  |  |  |  |  |  |  |  |  |  |  |  |  |
|  |  |  |  |  |  |  |  |  |  |  |  |  |  |  |  |  |  |  |  |  |  |  |  |  |  |  |
