# Supplementary material for: Xylella fastidiosa subsp. pauca and olive produced lipids moderate the switch adhesive versus non-adhesive state and viceversa
Source: PLoS One. 2020 May 15;15(5):e0233013. doi: 10.1371/journal.pone.0233013 (PMC7228078; doi:10.1371/journal.pone.0233013)
Supplement: Attachment — Submitted filename: 021020 PLOSE ONE ms review comments MN NPK.docx [file pone.0233013.s010.docx]

Review comments

Summary: In the current submitted manuscript, the authors described their work in profiling the lipids of *Xylella fastidiosa*, both from infected olive trees and the uninfected counterparts. Furthermore, they identified free fatty acids derivatives that are differentially represented in these two groups, and some of those inhibit or stimulate the growth of *X. fastidiosa.*

Comments:

Line 96. It was unclear which part of the plant was collected and how.

Line 101. Can authors elaborate further what they mean by “six pools from OQDS+ and six from OQDS-“? Does it mean that 10 samples from each group were pooled together? How many samples (or pools) then were subjected to the lipidomic analysis?

Line 156. Are most of the symptomless samples free of Xf, and are the OQDS+ samples mostly infected by Xf? Please provide a summary of OQDS and Xf PCR screen results

Line 159. The authors described two principle components in Figure 2-do both PC’s differentiate the Xf+ and Xf- samples? Please clarify.

Line 162. Please elaborate the reasons for selecting the 90 compounds for further analysis. What previous results specifically were the authors referring to? Please also provide a table with fold change and p-values listed for these 90 compounds.

Fig 1. Statistical corrections be taken into account for multivariant analysis. A more stringent cut-off should be applied.

Line 165. In Fig 3 the authors showed 10 lipids that are differentially abundant between the Xf+ and Xf- samples. It was unclear why these 10 lipids were chosen, so please elaborate further. Are they the most different ones? Did these show the biggest fold change?

Line 182, Fig 3. The authors said “The Y-axis shows the normalized relative abundance” though it is not clear how the normalization was done, and the axes appeared arbitrary. Please elaborate how these numbers were calculated.

Line 188. Even though other studies have shown SEMs of *Xylella fastidiosa* infecting other plants and forming biofilm (Newman et al., 2004), it was not confirmed that they do the same in the host being studied here. Can the authors confirm with a subset of Xf+ samples that biofilm was indeed formed? This will provide basis for linking lipid profiles with planktonic/biofilm growth.

Line 192. It was not shown that 7,10-diHOME were different between the Xf+ and Xf- samples, so why was this tested? Why was a mixture of 7,10-diHOME and 10-HOME tested? Minor point here: 9-HODE induced biofilm formation was shown in Fig 5, not Fig 4.

Line196. Can authors provide statistical support for the effect of these compounds shown in Fig S2?

Decision:

The study showed streamlined methods in lipid profiling of Xf infected plants from the field, which provides insights into the disease in the field. However, the basis for doing bacterial growth assays was not well elaborated and it was unclear why certain assays and conditions were used. Parameters and findings from the lipidomic analysis were not well interpreted. I suggest the authors address these points through data analysis and assessment of bacterial infection/burden/biofilm formation in the Xf+ samples before resubmission.
