# Supplementary material for: Xylella fastidiosa subsp. pauca and olive produced lipids moderate the switch adhesive versus non-adhesive state and viceversa
Source: PLoS One. 2020 May 15;15(5):e0233013. doi: 10.1371/journal.pone.0233013 (PMC7228078; doi:10.1371/journal.pone.0233013)
Supplement: Attachment — Submitted filename: 042120 PlosOne review comments.docx [file pone.0233013.s012.docx]

Please see additional reviewer’s comments in red:

Line 96. It was unclear which part of the plant was collected and how.

The description of how samples collection was performed is now added to the

manuscript at L72-77

Line 101. Can authors elaborate further what they mean by “six pools from OQDS+

and six from OQDS-“? Does it mean that 10 samples from each group were pooled

together? How many samples (or pools) then were subjected to the lipidomic analysis?

10 samples (derived from 10 different olive tresses) were pooled together and 12

samples were analysed.

Lipidomic analysis was performed on 12 pools for a total of 120 plants (60 infected and

60 non-infected); each pool was made putting together samples harvested from 10

plants. The sentence has been modified accordingly at L78-80

Line 156. Are most of the symptomless samples free of Xf, and are the OQDS+

samples mostly infected by Xf? Please provide a summary of OQDS and Xf PCR

screen results.

The real time PCR results are reported in this answer and, now, in S1 Figure. The

figure S1A shows the results of OQDS- samples, the figure S1B the results of OQDS+.

A)

A) B)

Samples were amplified following the protocol published by Harper and colleague [29].

Line 159. The authors described two principle components in Figure 2-do both PC’s

differentiate the Xf+ and Xf- samples? Please clarify. Are all OQDS+ samples Xf+, and OQDS- samples Xf-? I think by pooling the samples, any a pool of Xf+ samples could have Xf- samples that won’t be detected.

The PC1 distinguish Xf+ from Xf-. PC2 is more related to the type of lipids present as

highlighted in the corresponding loading plot. From this, we can suggest that specific

type of lipids are hallmarks of infection (e.g. oxylipins), some others are probably more

related to specificities in lipid composition of the different pools.

Line 162. Please elaborate the reasons for selecting the 90 compounds for further

analysis. What previous results specifically were the authors referring to? Please also

provide a table with fold change and p-values listed for these 90 compounds. accept this

The select compounds emerged from the previous dataset published in Scala et al.,

2018 doi https://doi.org/10.3389/fmicb.2018.01839 [30], and from selecting compounds

on the upper right and left box within the volcano plot of Figure 1 straight from mass

profiler professional output. Then, on these 90 compounds we performed product ion

fragmentation to confirm the ToF analysis. On those positively identified (~80%) we

performed MRM and SIM analysis to quantify them. Within this dataset (Table S2), only

25 were revealed within our samples (both Xf- and Xf+) and consequently quantified by

MRM (now shown in Table S3). 10 out of 25 resulted significantly different between Xf+

and Xf- samples (T Test and volcano plot in Table S3) and were used for building up

the boxplots of Figure 3.

Fig 1. Statistical corrections be taken into account for multivariant analysis. A more

stringent cut-off should be applied. Good

We worked on default settings of the mass profiler version (Cut-offs were 2 fold-change

and 0,05 p-value) working downstream, but knowing that this analysis could be more

stringent we chose almost half of the emerged compounds from the volcano plot and

specifically those presenting the highest FC and corrected P value.

Line 165. In Fig 3 the authors showed 10 lipids that are differentially abundant between

the Xf+ and Xf- samples. It was unclear why these 10 lipids were chosen, so please

elaborate further. Are they the most different ones? Did these show the biggest fold

change?

Among the set of 90 targets, we chose the most different between the Xf+ and Xf- samples.

The 10 compounds are the statistically significant ones (T-test, p-value<0,05;

Table S3) among targeted analysis tested compounds (listed in MRM table in Material

and Methods), as also stated above at answer at lane 162 question.

Line 182, Fig 3. The authors said “The Y-axis shows the normalized relative

abundance” though it is not clear how the normalization was done, and the axes

appeared arbitrary. Please elaborate how these numbers were calculated.

Target compounds’ peak areas were divided by their internal standard’s peak area and

Then by the maximum value of internal standard peak area

Line 188. Even though other studies have shown SEMs of Xylella fastidiosa infecting

other plants and forming biofilm (Newman et al., 2004), it was not confirmed that they

do the same in the host being studied here. Can the authors confirm with a subset of

Xf+ samples that biofilm was indeed formed? This will provide basis for linking lipid

profiles with planktonic/biofilm growth. Good

Unfortunately, we cannot confirm the biofilm formation in Xf+ olive samples, but we are

supported by Cardinale et al 2018 [34] who demonstrated that Xf form biofilm into

symptomatic olive trees cultivar (“Ogliarola di Lecce”). The cultivar used in this study

was the same we employed. Cardinale et al 2018 evaluated the role of bacterial

aggregates in vessel occlusions and quantified the level of infection and vessel

occlusion in both petioles and branches of naturally infected and non-infected olive

trees. The authors demonstrated that symptomatic petioles showed colonization by X.

fastidiosa, and the vessels appeared completely occluded by a biofilm containing

bacterial cells and extracellular matrix. Thus, Cardinale and colleagues point out the

primary role of the pathogen in olive vessel occlusions and underline the bacteria role

in causing vessel occlusion in leaves. The paper is now cited in ref section [34].

Line 192. It was not shown that 7,10-diHOME were different between the Xf+ and Xfsamples,

so why was this tested? Why was a mixture of 7,10-diHOME and 10-HOME

tested? Typo: figure 3 y-axis legend 10-HpOME. was 7,10-diHOME and 10-HOME detected in your LC-ToF and LC-MS data? Did they show any difference? *P. aeruginosa* has a diol synthase operon that’s regulated by a regulatory unit encoded downstream. does Xf have orthologs of these genes in a similar genomic architecture?

The oleic-derived oxylipins present in figure 3 is the 10-HpOME that is a relatively

unstable product and thus difficult to provide in vitro. Following the study of Martinez et

al. 2016 [24], 10-HpOME is readily converted to 7,10-diHOME and 10-HOME. These

products are commercially unavailable, and we asked the Martinez group to provide

them and they kindly send us these oxylipins. Unfortunately, 10-HOME is in mixture

with 7,10-diHOME and the 7,10-diHOME was pure. We inserted them in the in vitro

tests since they are, as indicated in Martinez et al 2016 and 2018 [24, 25], part of the

oleic acid oxylipin pathway and a quorum-sensing molecule in Pseudomonas

aeruginosa; furthermore, in Scala et al. 2018 [30] we found 7,10-diHOME in Xf, thus in

this manuscript we aim at verifying if this compound could act in Xf similarly to P.

aeruginosa. Scala et al. 2018 found 7,10-diHOME < LOQ in vitro conditions

Minor point here: 9-HODE induced biofilm formation was shown in Fig 5, not Fig 4.

Concerning Fig. 5, we suppose it is due to the consequent numeration generated by

the PloS submitting system. Thus, the boxplots are reported in the second page of the

figure 4.

Line196. Can authors provide statistical support for the effect of these compounds

shown in Fig S2?

Tested compounds were evaluated through Kruskal Wallis test and classified by Fisher

LSD post-hoc test with Bonferroni correction (p-value<0,05).

Discussion:

The results provided in the study are convincing that Xf infection is associated with the lipidomic profiles of olive petiole samples. It should be cautioned that such comparison didn’t consider the growth stage of Xf, and presumably, the Xf+ samples would be undergoing different stages of Xf growth. Though the different effects of these lipids on Xf planktonic and biofilm growth were intriguing, a time course that captures different stages of Xf growth in vivo is needed to provide the biological significance of the changed lipid profile and the actions of specific lipids.

It should also be considered that increased lipid abundance in Xf+ samples could result from induction of bacterial metabolite (as shown in the P. aeruginosa study that the authors referred to) or plant defense (both 9-HODE and 13-HODE are known oxylipins for plant defense).

Comments: The authors addressed our questions through detailing the rationales behind the study and supplementing data and methods that were previously missing or poorly explained. This overall meets the expectation. I accept this manuscript if the authors address a few points highlighted here in red.
